# Photosynthetic Efficiency and Yield of Cucumber (*Cucumis sativus* L.) Grown under HPS and LED Lighting in Autumn–Winter Cultivation

**DOI:** 10.3390/plants10102042

**Published:** 2021-09-28

**Authors:** Janina Gajc-Wolska, Katarzyna Kowalczyk, Arkadiusz Przybysz, Małgorzata Mirgos, Paweł Orliński

**Affiliations:** 1 Department of Vegetable and Medicinal Plants, Institute of Horticultural Sciences, Warsaw University of Life Sciences WULS-SGGW, Nowoursynowska 159, 02-776 Warszawa, Poland; janina_gajc_wolska@sggw.edu.pl (J.G.-W.); malgorzata_mirgos@sggw.edu.pl (M.M.); krwl@sggw.edu.pl (P.O.); 2 Department of Plant Protection, Institute of Horticultural Sciences, Nowoursynowska 159, 02-776 Warsaw, Poland; arkadiusz_przybysz@sggw.edu.pl

**Keywords:** gas exchange, light quality, net carbon assimilation, photochemical activity of the photosystem II, SPAD index

## Abstract

The objective of this study was to evaluate the effect of the supplemental lighting of cucumber with sodium pressure lamps (HPSs) and light-emitting diodes (LEDs) on photosynthetic efficiency and yield in autumn–winter cultivation. Cucumber plants of the ‘Svyatogor’ F1 midi-cucumber parthenocarpic type cultivar were grown on mineral wool mats in three compartments, differing only in the type of light, i.e., (1) HPS top lighting (HPS) in the first compartment, (2) HPS top lighting and LED panel interlighting (HPS + LED) in the second compartment and (3) LED top lighting and inter-row LED panels (LED) in the third compartment. The photosynthetically active radiation was the same in each compartment. The study showed that the highest commercial yields of cucumber could be achieved under LED light (top and inter-row). The chlorophyll content in the leaf blade of younger leaves was higher in plants under LED lighting. This type of lighting also had a positive effect on the gas exchange of plants (net carbon assimilation, stomatal conductance, transpiration). LED and HPS + LED lighting increased the chlorophyll *a* fluorescence parameters, such as Fs, Fm’ and vitality index (PI), in both younger and older leaves, which also increased the fruit yield in the tested combinations.

## 1. Introduction

Natural light is an essential source of energy for plant growth and development. In countries in the north of the European continent, there is a strong deficit of natural light during autumn, winter and early spring, which significantly limits plant growth. Therefore, during this period, it is necessary to introduce an additional artificial light source for plant lighting [1]. Many different types of lamps with a wide emission spectrum in the PAR (*photosynthetically active radiation)* range are used for this purpose. A common feature of these lamps is that they are very different spectrally compared to sunlight, which significantly affects plant morphology [2]. It was indicated that light-emitting diodes (LEDs) are the optimal light source for plants [1,3,4,5]. LEDs can provide freely adjustable wavelength specificity in order to optimize leaf photosynthesis in crop production [6]. Lighting installations, both as top and intercrop lights, are frequently utilized in cucumber and tomato cultivation because of the higher yields associated with these lighting systems [7,8]. Currently, the lamps installed in both positions are of the HPS (*high-pressure sodium*) type. Sodium lamps are still the most commonly used lights in greenhouse vegetable production because they efficiently light plants from above. The light spectrum of sodium lamps is determined by their design and, therefore, is difficult to modify. The spectrum of sodium lamps is characterized by a low proportion of blue light. Plants require complex light for optimal growth and development, but the mechanisms of plant responses to light are still poorly described and not fully understood [9]. According to Liu et al. [9], the supplementation of red and blue light with green and white light promotes the growth of rapeseed seedlings in a controlled environment. Brazaityte et al. [10] recorded a beneficial effect of supplementary green light in composite light on the growth and quality parameters of cucumber. Adding green and yellow light wavelengths to red and blue LEDs had a positive effect on lettuce morphology and dry matter accumulation [11]. The growth and quality of lettuce and other leafy vegetables were shown to be the greatest when the light use efficiency was the highest. This was obtained at a light intensity of 200 µmol m^−2^ s^−1^ and a 25–50% share of blue light compared to red light [12]. Furthermore, it was reported that lettuce grown under low-intensity red and blue LEDs with a 50% share of blue light demonstrated a higher light-use efficiency compared to white light and other light sources [13]. 

During photosynthesis, plants use a spectrum of light in the blue (400–500 nm) and red (600–700 nm) color ranges. Some reports indicated that the use of a single red or blue LED light source or their combination could improve the efficiency of photosynthesis to promote plant growth and regulate morphogenesis [14,15,16]. Blue light strongly affects plant growth and development, including leaf size, stomatal opening and photosynthesis. Regarding the high photosynthetic capacity at the chloroplast level, it was found that the plant response to blue light is similar to the plant response to natural light. Plants typically show higher photosynthetic intensity under blue light than under red light [17]. Previous studies on the spectral quantum efficiency of leaf photosynthesis showed that it is highest under a red spectrum [18,19,20]. However, the short-term result obtained with red light does not give the optimal photosynthetic intensity in the long term. Cucumber plant leaves grown under red LED light (100 μmol m^−2^ s^−1^ at 640 nm) showed low Fv/Fm dark adaptation [21], indicating photo-damage to the leaf blade [22]. Plant growth under red light only is characterized by a lack of leaf response to stomatal movement, low photosynthetic intensity, reduced nitrogen uptake, low dry matter and impaired plant development [21]. In contrast, the introduction of blue light at the same intensity as red light significantly increases photosynthetic intensity, chlorophyll content and dry matter in species such as rice [23], spinach [24] and cucumber [21]. Despite the many studies conducted to date, the effects of blue and red light on specific photosynthetic processes remain largely unclear. Photochemical processes in the plant primarily respond to light quality and light changes in the environment by converting solar energy to chemical energy by photosystems II and I (PSII and PSI) [25]. PSII was found to be sensitive to light quality. Blue light can lead to maximally efficient photochemical quantum yields of PSII (Fv/Fm and ΦII), whereas red light inhibits such processes [2]. Light-quality-induced variation in PSII activity may affect PSI activity. PSI plays a key role in photochemical processes by transporting electrons from PSII, as well as controlling the cyclic electron flow [26]. Given that PSII and PSI act electrochemically in series, any excitation imbalance between the two photosystems can affect the linear electron flow [27,28]. Studies have shown that light quality affects the ratio of PSII to PSI in plants such as cucumber [29], pea [30] and thale cress [27] in vivo or in vitro. Decreased photosynthesis due to different stress conditions reduces crop productivity. 

The phenomenon of chlorophyll fluorescence is used to determine the efficiency of the photosynthetic apparatus and the overall health of the PSII in plants [31,32,33]. Measurements of chlorophyll *a* fluorescence parameters provide detailed information on the state of the photosynthetic apparatus and damage within the chloroplasts. They also help to estimate the effects of environmental stresses on plant growth and yield [34]. Two parameters related to chlorophyll *a* fluorescence, namely PI (*performace index*) and Fv/Fm, are most commonly used as indicators of stress susceptibility. PI reflects photosystem II functioning and is a measure of plant performance under stress conditions, while the parameter Fv/Fm estimates the maximum quantum yield of PSII [35,36]. The assessment of chlorophyll *a* fluorescence can therefore be a useful tool to evaluate the energy and metabolic balance of photosynthesis and the level of productivity of different plant species under stress conditions, which may be blue and red lighting of plants [37,38,39,40]. Cucumber (*Cucumis sativus* L.) is one of the most important vegetables grown under cover worldwide. It is a typical heliophile that is sensitive to light quality [16]. The current state of knowledge on the physiological, metabolic and genetic response of cucumber to LED lighting is insufficient. The search for the response of individual cucumber cultivars to LED lighting and their possible adaptation to changing conditions may allow for the selection of cultivars with increased adaptability and higher tolerance to blue and red LED lighting. The aim of the study was to evaluate the effect of greenhouse cucumber lighting on yield and selected gas exchange and chlorophyll *a* fluorescence parameters of cucumber under different light sources.

## 2. Results and Discussion

### 2.1. Effects of Supplemental Light with HPS and LED Lamps on the Yield in Different Months of Autumn–Winter Cultivation

The obtained results indicated that the differentiated light sources had a significant effect on the yield of cucumber plants. The highest marketable yield was characterized by plants lighted by LED lamps during the whole cultivation cycle (from the beginning of September to the end of April). A lower yield, especially in the months from September to December, was obtained from plants lighted under HPS + LED lamps and HPS lamps. In the following months of cultivation, from January to April, considering the combinations HPS + LED and HPS, the plants under supplementary HPS + LED lighting produced a higher yield compared to the yield of plants that were grown under the HPS lamps. 

Taking into account the individual months of cultivation, it was observed that in each month of cultivation, a significantly higher yield was characteristic for plants from the LED combination. It is also worth noting that despite the lighting, natural light played a key role in the yield volume. In the months with low irradiation intensity (November, December and January), the yield was at a significantly lower level than in the remaining months, where the effect of natural light on the amount of obtained marketable yield of cucumber plants was marked (Table 1). According to Kleniewska et al. [41], the monthly averaged daily diffuse solar radiation in Poland during these months is 1.35, 1.30 and 1.25 MJ m^−2^ respectively.

The results described above regarding the obtained marketable yield of cucumber from the three tested combinations were reflected in the measurement of the chlorophyll content and selected gas exchange and chlorophyll *a* fluorescence indicators for younger and older leaves.

### 2.2. Effects of Supplemental Light from HPS and LED Lamps on Chlorophyll Content in Young and Old Leaves

The chlorophyll content in leaves is one of the most important factors determining photosynthetic capacity. This study showed that the obtained results of chlorophyll content in younger and older cucumber leaves were at a similar level, but the amount of pigments depended on the type of lamps and the measurement date (Table 2 and Table 3). Significantly higher chlorophyll content was found in young leaves lighted with LED lamps in November, December and March and in leaves lighted with HPS + LED lamps in December and March compared to leaves grown under HPS lamps at all measurement dates (Table 2). By observing the results of the measurement of chlorophyll content in older cucumber leaves, it was found that leaves lighted with all types of lamps in November and December were characterized by a similar value of this index, while in March, significantly higher chlorophyll content was recorded in leaves supplemented with LED and HPS + LED lamps compared to leaves lighted with HPS lamps, which was due to the fact that in March, the intensity of natural light irradiation was high. This led to the intensive growth of the leaf blade of the upper leaves, and the lack of inter-row irradiation caused a decrease in the chlorophyll content of older cucumber leaves (Table 3). The results of previous studies indicated that light quality, but mainly blue and red light color, has a significant effect on the photosynthesis and photomorphogenesis of leaves, as well as on plant physiology [2,17]. The cucumber plants that grew under LED lamp lighting were characterized by more compact growth and slightly smaller leaf blade area but thicker tissue. This contributed to an increased chlorophyll content compared to the leaves of plants lighted with HPS lamps, which had a large leaf blade area, thin tissue and, thus, a slightly lower chlorophyll content.

### 2.3. Effects of Supplemental Lighting with HPS and LED Lamps on Photosynthetic Characteristics of Young and Old Leaves

**Figure 1:**
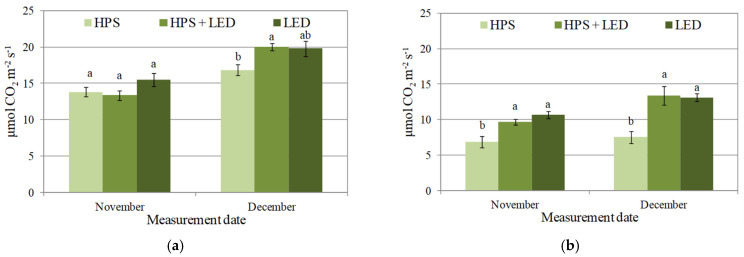

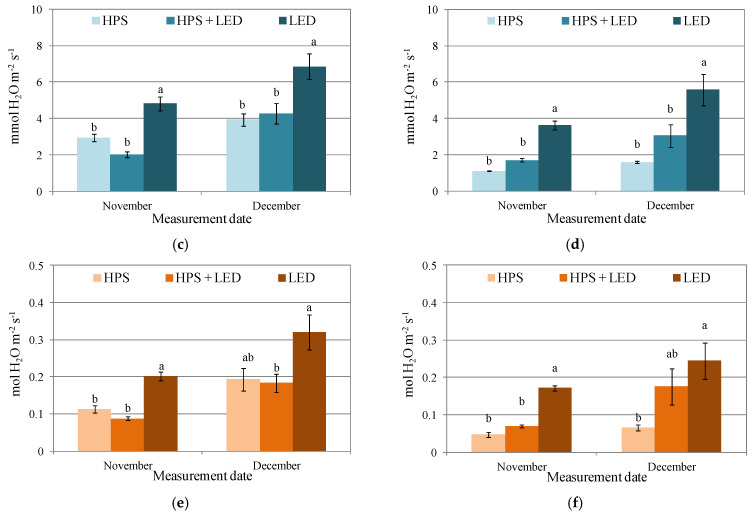
Effect of different light sources (HPS, HPS + LED, LED) on net carbon assimilation ((**a**) young leaves, (**b**) old leaves), transpiration rate ((**c**) young leaves, (**d**) old leaves) and stomatal conductance ((**e**) young leaves, (**f**) old leaves) in cucumber plants. Data are given as mean ± SE, *n* = 5. Different lowercase letters indicate a significant difference at *p* < 0.05 using Tukey’s test.

The type of light influenced the intensity of gas exchange in the cucumber plants (Figure 1a–f). Regardless of the date of measurement and age of the leaf, the net carbon assimilation was the lowest (usually significantly so) in plants lighted with HPS lamps. The highest value of net carbon assimilation in November was recorded for plants exposed to LED lights, while in December, similar results were noted for plants grown under LED lights and a combination of HPS and LED lights (Figure 1a,b). The transpiration rate was always significantly higher in plants lighted with LED lights (Figure 1c,d). The high net carbon assimilation and transpiration rate in the examined leaves exposed to LED lights resulted from significantly higher stomatal conductance (Figure 1e,f). 

The higher proportion of blue light in the spectrum of LED lamps compared to sodium lamps, with the same light intensity and illumination length in all tested combinations (HPS, HPS + LED, LED), could have been the reason for the greater efficiency of light assimilation by cucumber plants. It should also be noted that the inter-row LED lamps, providing extra light to the plants, were probably also of great importance for the higher yield. The positive effect of blue light on stomatal conductance was already recorded by Sharkey and Raschke [42]. Increased stomatal conductance ensures an easier and greater CO_2_ flow to the chloroplast, thus more efficient photosynthesis. Blue light from LEDs may also positively affect the stomatal length, width and opening; stomata number per area; and total stomatal pore area index [43]. It is a well-known phenomenon that the light spectrum affects gas exchange and photosynthesis [2,17,44]. Savvides et al. [17], similar to the results obtained in this work, showed that the presence of blue light in LED lamps had a positive effect on the intensity of gas exchange and, consequently, photosynthesis in cucumber plants. Higher net carbon assimilation in plants of different species (*Pterocarya*, lettuce, cherry radish, cherry tomato, einkorn) was recorded after treatment with blue light [43] and different combinations of blue and red light [45,46]. On the other hand, Lanoue et al. [47] showed that the photosynthetic rate was similar irrespective of the light spectrum, while Yang et al. [48] demonstrated that the net photosynthetic rate of plants lighted with blue light was lower compared to other light treatments. 

In order to evaluate how the type of lamp affects the gas exchange induction in cucumber plants, the net carbon assimilation was measured for 20 min after turning on the lamps. For the first seven minutes after the lamps were turned on, the net carbon assimilation was the highest; however, this was not always significantly so in plants lighted with HPS (Table 4). The obtained results indicated that plants lighted with HPS lamps were characterized by a faster start of photosynthesis. However, from the eighth minute of lamp operation, when the net carbon assimilation stabilized at a relatively constant level, the highest (not always significantly) values of this parameter were recorded for plants exposed to LED light, followed by those lighted with a combination of HPS and LED lights (Table 4).

### 2.4. Effects of Supplemental Lighting with HPS and LED Lamps on Chlorophyll a Fluorescence of Young and Old Leaves

Chlorophyll *a* fluorescence is a method for studying abiotic and biotic stress factors affecting the functioning of the photosynthetic apparatus. The application of this method provides a lot of information about the vegetation of the plant. It allows for observing the initial symptoms of plant stress even before the visible reduction of chlorophyll content in the leaves [49]. The results obtained indicate that the value of the basic fluorescence index of chlorophyll *a* Fs (fluorescence constant of the sample adapted to light) was different depending on the leaf age, light source and measurement date (Table 2 and Table 3). Taking into account the measurement of the Fs of young leaves, the highest value of this index (over 600 a.u.) was characteristic for leaves under LED light in December, while the lowest value was obtained for leaves under HPS light in March (slightly over 400 a.u.) (Table 2). Similarly, the highest value of the Fs index was characterized by older leaves lighted with LED lamps in November (more than 500 a.u.), while the lowest index was obtained for leaves under HPS light in March (less than 300 a.u.) (Table 3).

The results of this measurement indicate that the appropriate ratio of blue to red light that is possible with LED lamps favors photosynthesis in cucumber leaves compared to HPS lamps, which are characterized by high red and infrared light intensity and low blue light intensity [44]. In their experiment, Savvides et al. [17] showed that a combination of blue and red light in a ratio of 30:70 resulted in increased photosynthetic efficiency compared to monochromatic light. As mentioned earlier, the lack of inter-row lighting of the plants in the combination with HPS lamps significantly reduced the results of the solid fluorescence measurements for older cucumber leaves on all measurement dates. 

An important indicator that shows the functioning of the photosynthetic apparatus is the maximum fluorescence of the sample adapted to light (Fm’). The results obtained show that differences in the value of this parameter depended on the age of the leaf, the light source and the time of measurement (Table 2 and Table 3). It was found that the highest value of Fm’ (more than 2300 a.u.) was obtained for younger leaves lighted with HPS + LED and HPS lamps in November and with LED lamps (more than 2300 a.u.) in December, while a lower value of this index was characteristic of leaves supplemented with all types of lamps in March (Table 2). For older leaves, the value of fluorescence of the maximum sample adapted to light (Fm’) was higher when lighted with HPS + LED and LED lamps on the three measurement dates (about 2300 a.u.), while it was lowest on all measurement dates for leaves supplemented with HPS lamps (Table 3). The adaptation of young leaves to LED light was slightly different than the adaptation to light from HPS lamps, as can be seen in Table 2.

Young cucumber leaves grown under LED lamps required longer adaptation to achieve maximum fluorescence compared to leaves under light from HPS lamps. The reaction of older leaves was slightly different. The inter-row irradiation resulted in a higher maximum fluorescence value of the cucumber leaves compared to the combination with HPS top lighting only.

Another parameter of chlorophyll *a* fluorescence illustrating the physiological state of the plant is the current quantum yield (PSII). Analysis of the PSII parameter measurement results showed that both younger and older leaves obtained a high value of this index, which indicates the efficient functioning of the photosynthetic apparatus (Table 2 and Table 3). A significantly higher value of PSII was recorded in young leaves lighted with LED and HPS + LED lamps in November and December (0.79 a.u.), while the lowest value was recorded in leaves supplemented with HPS lamps, also in November and December (0.68 and 0.72 a.u., respectively). In March, the value of the PSII index was at a similar level for each lamp type (0.75 a.u.) (Table 2). The value of the current quantum efficiency index (PSII) in old leaves was at a similar level regardless of the type of lamp used and the date of lighting, and ranged from 0.76 to 0.81 a.u. (Table 3).

One of the most commonly used parameters for measuring chlorophyll *a* fluorescence is the parameter of maximum quantum yield (Fv/Fm), which measures the proportion of absorbed light by chlorophyll PSII molecules that are used during photochemical processes, i.e., it estimates the amount of photosynthesis [50]. The results obtained show that the value of this parameter was at a similar level for both younger and older leaves (Table 2 and Table 3). 

Analyzing the results of the measurement of the maximum quantum yield (Fv/Fm) for younger and older leaves, it was found that leaves lighted with each type of lamp (LED, HPS + LED, HPS) in three measurement terms obtained a similar value of this parameter. It ranged from 0.79 to 0.83 a.u. according to Bjorkman and Demmig [51] and this is the optimum value for most higher plants obtained under physiological conditions. Both the measurement values of current quantum yield and maximum quantum yield indicated that the photosynthetic apparatus of plants maintained the high PSII quantum yield of younger and older cucumber leaves, as observed in wheat leaves [52].

The results of the study also showed significant differences in the vitality index (PI) values for younger and older leaves depending on the light source and measurement date (Table 2 and Table 3). 

For younger leaves lighted with all types of lamps (LED, HPS + LED, HPS), the value of this index was at a similar level on the two measurement dates (November and December), while in March, there was a significant increase in PI value for leaves under LED lamps. The value of this index also increased for leaves lighted with HPS + LED and HPS lamps, but was significantly lower than the PI value obtained for leaves lighted with LED lamps (Table 2). The analysis of the vitality index (PI) values of older leaves showed that leaves lighted with LED and HPS + LED lamps in November and March obtained higher PI values, while in December, the value of this index decreased. It is worth noting that the vitality index developed quite differently for leaves supplemented with HPS lamps. In all measurement months, this index had almost the same values (close to 6) and only in December did the PI values not differ significantly irrespective of the type of lamps used. In the remaining months, the difference in vitality index values between plants lighted with HPS lamps and LED and HPS + LED lamps was statistically significant (Table 3). A similar response of the PI index of cucumber leaves to varying light quality was obtained in their study by Tikkanen et al. [53].

## 3. Materials and Methods

### 3.1. Experimental Conditions 

**Figure 2:**
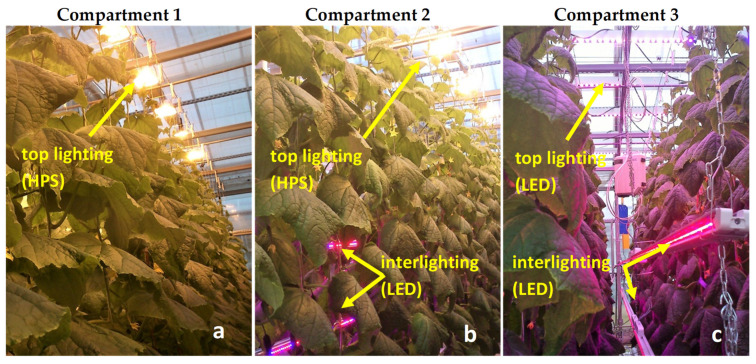
Experiment lighting schemes: (**a**) HPS, (**b**) HPS + LED and (**c**) LED.

The experiment was carried out in autumn–winter 2015–2016 in a greenhouse at the Warsaw University of Life Sciences (longitude 21° E, latitude 51°15′ N) as a part of a scientific project conducted with Philips Lighting Holding B.V. Three experimental growing compartments, each with a usable area of about 40 m^2^, were equipped with assimilation lighting lamps (the technical equipment of the cultivation rooms and the cultivation parameters for cucumber were the same as for the research described in the article by Kowalczyk et al. [54]). In the study, a compartment with conventional overhead lighting by Gavita GAN 600 W (200 W m^−2^) HPS lamps was the control (HPS). In the next compartment, inter-row LED panels were installed with the HPS 600 W (150 W m^−2^) top lighting, with 2 panels installed horizontally between plants, each panel emitting 50 µmol m^−2^ s^−1^ PPFD (photosynthetic photon flux density; Philips Green Power LED module 2.5 m HO DR/B 100 W), as a combination (HPS + LED). In the third compartment, Philips Green Power LED top lighting units (DR/W—LB, 195 W) were installed as top lighting, obtaining 220 µmol m^−2^ s^−1^ PPFD and inter-row LED lamps in 2 panels were installed horizontally between plants (Philips Green Power LED module 2.5 m HO DR/B 100 W), as a combination (LED). The LED lamp characteristics were 87.5% red light in the wavelength range of 630 to 660 nm, with the highest proportion at 660 nm and 12.5% blue light in the range of 440 to 460 nm. The experimental lighting schemes are presented in Figure 2.

By measuring PAR with a Li-Cor Light meter LI-250A, quantum sensor LI-190 with all lamps on, an ~320 µmol m^−2^ s^−1^ PPFD (photosynthetic photon flux density) was obtained in each compartment. In the growing compartments, the microclimate was computer-controlled: 18 h day setting, where the lamps were turned off when the natural light intensity reached the level of 300 W m^−2^ and when the internal temperature exceeded 30 °C. The inter-row lighting worked independently of the conditions. Cucumber ‘Svyatogor’ F1, which is a midi-cucumber parthenocarpic type cultivar (Rijk Zwaan), of high shade tolerance and 18–22 cm fruit length, was the model plant. Seeds were sown on 12 August. Seedlings were produced in rockwool blocks under the following conditions: temperature of 22 °C during the day/20 °C at night, soil humidity of 70–80%, air humidity of 70% and light intensity of daylight with supplementary HPS light at 170 μmol m^−2^ s^−1^. Cucumber plants were transplanted to the rockwool slabs in the three growing compartments 23 days after sowing (DAS). Cucumber plants were grown on Grodan mineral wool mats. The plants were managed using the high wire method. Twice a week, all side shoots and clinging tendrils were removed from the fruiting shoots, as well as the 3 oldest leaves of each plant. The plants were lowered diagonally. Regulation of the number of buds per shoot was also carried out, maintaining a balance between generative and vegetative plant development. The nutrient solution for fertigation was based on one-component fertilizers where each 1 dm^3^ volume contained 195 mg of N in the form of NO_3_ and 6 mg in the form of N-NH_4_; the following elements (in mg) were also present: P—56, K—264, Mg—44, Ca—257, Fe—2.47, Mn—0.83, B—0.68, Cu—0.15, Zn—0.14 and Mo—0.08. The pH and EC values were monitored during the plant growth and maintained at the average levels of pH 5.7 and 3.2 mS cm^−1^ EC, respectively. The nutrient solution was dosed into the plant through a capillary system, with one capillary for each plant. The dosage of the nutrient solution was controlled using a fertilization computer.

The EC value in the substrate ranged between 3.2 and 4.0 mS cm^−1^. Microclimate parameters in growing compartments were controlled using a Synopta Climate Computer and were as follows: temperature was about 22−25 °C/18−22 °C during the day/night, the CO_2_ concentration was supplied up to the level of 800 ppm and the RH of the air was at the level of 70%.

### 3.2. Fruit Yield

Fully grown fruit weighing about 200–210 g was harvested daily. The fruit harvest started on 43 DAS. Differences in cucumber fruit yield from the individual growing months were determined by the different plant lighting.

### 3.3. SPAD Index, Gas Exchange and Chlorophyll a Fluorescence Measurements

Six plants in each of the three experimental compartments were randomly selected for the study. Chlorophyll (SPAD index), gas exchange and chlorophyll *a* fluorescence were measured for each plant at the 5th fully grown leaf from the top of the plant (younger leaf) and at the 10th leaf (older leaf). The single plant was a biological repetition and all measurements were performed once a week in each of the three chosen months of cucumber cultivation. These measurements were made, depending on the method of lighting of the plants and the age of the leaves, in individually chosen growing months of the year: November, December and March. 

The following measurements were taken from fully fruiting plants: 

Chlorophyll (SPAD index) was estimated using the portable equipment SPAD-502 (Minolta, Japan) in the same leaves in which the fluorescence was measured.

Gas exchange (net carbon assimilation (μmol CO_2_ m^−2^ s^−1^), stomatal conductance (mol H_2_O m^−2^ s^−1^) and transpiration rate (mmol H_2_O m^−2^ s^−1^) were measured with an LI-6400 Photosynthesis System (LI-COR, Inc., Lincoln, NE, USA) equipped with a 6400-40 Leaf Chamber Fluorometer and a 6400-01 CO_2_ mixer. Measurements were conducted between 9 am and 11 am, when changes in the environmental conditions were relatively small. Measurements were taken at the reference CO_2_ (450 µmol s−^1^) with a constant flow rate (400 µmol s^−1^), relative humidity ranging from 30 to 50%, constant photosynthetic photon flux density (PPFD, 8000 µmol m^−2^ s^−1^) and constant temperature (30 °C). Measurements were carried out on two dates (November and December, 85 and 125 DAS respectively) on young (5th leaf from the top) and older leaves (10th leaf from the top) from 5 randomly selected plants (biological replication). Additionally, the net carbon assimilation was measured for the first 20 min after turning on the lamps (results were recorded every 60 s). Measurements were carried out for 3 consecutive days in November, 100 DAS.

Chlorophyll *a* fluorescence was measured with the use of the FMS-2 Field Portable Pulse Modulated Chlorophyll Fluorescence Monitoring System (Hansatech Instruments Ltd., King’s Lynn, Norfolk, England) and the following parameters were measured: Fs—steady-state fluorescence yield, Fm’—light-adapted fluorescence maximum and ‘PSII (Fv’/Fm’)—quantum efficiency of photosystem II (PSII). The Pocket PEA fluorescence meter (Hansatech Instruments Ltd., King’s Lynn, Norfolk, UK) was used to measure the direct fluorescence, obtaining a measurement of the maximum efficiency of the plant’s photosynthetic camera after an earlier 30 min of adapting the leaves to darkness (using special clips). The following parameters were analyzed: maximum efficiency of the PSII photosystem in the dark (Fv/Fm) and PI (PSII vitality index).

The cultivation day was started at night and the first measurement of the photosynthetic activity of the cucumber was studied during the time before and after the lamps were switched on to check how quickly the plant started to adapt to intense light under different lamp variants. 

### 3.4. Statistical Analysis

The data were subjected to one-factorial analysis of variance (ANOVA) using Statgraphics Plus 4.1 (Statpoint Technologies Inc., Warrenton, VA, USA). The differences between means of combinations were evaluated using the post hoc Tukey’s honestly significant difference (HSD) test. The means were considered to be significantly different when *p* < 0.05. The data are presented as mean ± SE. The number of samples for individual parameters is given in the descriptions for the relevant graphs and tables.

## 4. Conclusions

In this work, for the first time in greenhouse cucumber production, it was shown that supplementing sunlight with LED lighting and extra inter-row LED panels increased the efficiency of photosynthetic apparatus plants and, consequently, the yield of cucumber more than HPS light above or top sodium lamps with inter-row LED lamps.

The highest commercial yields in cucumber could be achieved with LED light (top and inter-row) throughout the growing cycle. The chlorophyll content in the leaf blade of younger cucumber leaves was higher when plants were supplemented with LED lamps. Furthermore, when plants were lighted between rows in the early spring months of cultivation, the chlorophyll content in the leaf blade of older leaves increased. The LED light also had a positive effect on plant gas exchange (net carbon assimilation, stomatal conductance, transpiration rate). 

The LED and HPS + LED light treatments increased Fs and Fm’ in both younger and older leaves (especially in the months of low natural light intensity), which also increased the fruit yield in the tested combinations. The obtained values of current quantum efficiency (PSII) and maximum quantum efficiency (Fv/Fm) indices for younger and older leaves lighted with different lamp types indicated that the photosynthetic apparatus of plants maintained a high quantum efficiency of PSII. LED and HPS + LED lighting increased the vitality index (PI) in both younger and older leaves of cucumber plants, with older leaves having a lower PI than younger cucumber leaves.

## Figures and Tables

**Table 1:** Effect of lighting type on cucumber fruit yields from September to April (kg m^−2^).

Month of Cultivation	Lighting Type			Means
	HPS	HPS + LED	LED	
September	4.34 c *	4.66 c	5.91 c	4.97 C *
October	11.46 b	13.00 b	16.03 a	13.49 B
November	11.04 b	12.46 b	13.72 b	12.40 B
December	7.20 bc	8.44 bc	8.53 bc	8.05 BC
January	4.87 c	10.30 b	11.18 b	8.78 BC
February	11.82 b	15.06 ab	15.93 a	14.27 AB
March	12.42 b	14.21 b	15.90 a	14.18 AB
April	14.08 b	15.56 ab	19.82 a	16.49 A
Means	9.65 B	11.71 AB	13.38 A	

* Different letters indicate a significant difference at *p* < 0.05 using Tukey’s test. Small letters indicate differences for the interaction: lighting type x month of cultivation. Capital letters indicate differences for the factors: lighting type and month of cultivation.

**Table 2:** Effect of lighting type and time of measurement on the value of the chlorophyll *a* fluorescence parameters and SPAD in young leaves.

Parameter	Time of Measurement	Lighting Type		
		HPS	HPS + LED	LED
SPAD	November	39.33 b *	35.97 c	46.05 a
	December	37.63 c	43.03 ab	43.47 ab
	March	42.35 ab	49.38 a	48.83 a
Fs	November	462.50 ab	590.50 a	617.33 a
	December	499.00 b	482.17 b	642.33 a
	March	418.50 ab	491.50 a	462.17 a
Fm’	November	2271.83 a	2315.00 a	2017.17 ab
	December	2079.83 ab	2077.33 ab	2346.83 a
	March	1919.33 b	1827.83 b	1878.00 b
PSII	November	0.681 b	0.743 a	0.793 a
	December	0.722 b	0.765 ab	0.760 a
	March	0.740 a	0.767 a	0.753 a
Fv/Fm	November	0.822 a	0.810 ab	0.797 b
	December	0.829 a	0.824 a	0.822 a
	March	0.818 ab	0.822 a	0.815 ab
PI	November	3.71 c	4.02 b	3.93 c
	December	4.03 b	4.08 b	4.23 b
	March	5.68 ab	5.87 ab	7.18 a

* Different lowercase letters indicate a significant difference at *p* < 0.05 using Tukey’s test.

**Table 3:** Effect of lighting type and time of measurement on the value of the chlorophyll *a* fluorescence parameters and SPAD in old leaves.

Parameter	Time of Measurement	Lighting Type		
		HPS	HPS + LED	LED
SPAD	November	46.42 ab *	45.50 b	46.88 ab
	December	44.93 bc	40.82 c	43.47 bc
	March	44.18 bc	48.83 a	53.92 a
Fs	November	409.83 b	510.33 ab	553.33 ab
	December	461.83 b	517.00 ab	502.17 b
	March	285.00 b	426.67 ab	386.00 ab
Fm’	November	1987.00 b	2240.17 a	2340.00 a
	December	2158.50 ab	2230.67 a	2292.33 a
	March	1567.33 c	2017.33 ab	2061.50 ab
PSII	November	0.772 ab	0.794 a	0.763 ab
	December	0.770 ab	0.784 a	0.779 a
	March	0.790 a	0.811 a	0.766 ab
Fv/Fm	November	0.836 a	0.830 a	0.825 a
	December	0.835 a	0.833 a	0.832 a
	March	0.829 a	0.829 a	0.827 a
PI	November	6.06 b	6.51 ab	7.38 a
	December	6.05 b	5.43 b	5.85 b
	March	5.87 b	6.88 ab	7.54 a

* Different lowercase letters indicate a significant difference at *p* < 0.05 using Tukey’s test.

**Table 4:** Effect of different light sources (HPS, HPS + LED, LED) on the net carbon assimilation measured during the first 20 min after turning on the lamps. Data are given as mean ± SE, *n* = 3.

Time from Turning on the Lamps (min)	Net Carbon Assimilation (μmol CO_2_ m^−2^ s^−1^)		
	Light Conditions		
	HPS	HPS + LED	LED
1	0.11 a * (±0.00)	−2.12 b (±0.15)	−2.46 b (±0.69)
2	2.24 a (±0.01)	−0.71 b (±0.05)	−0.51 b (±0.19)
3	4.74 a (±0.14)	1.72 c (±0.06)	2.71 b (±0.23)
4	5.66 a (±0.07)	3.27 c (±0.11)	4.99 b (±0.10)
5	7.29 a (±0.09)	5.87 b (±0.06)	6.86 a (±0.26)
6	7.55 a (±0.07)	6.64 b (±0.11)	7.43 a (±0.13)
7	8.95 a (±0.22)	8.11 a (±0.24)	8.22 a (±0.20)
8	8.84 a (±0.29)	8.86 a (±0.34)	9.02 a (±0.28)
9	8.99 a (±0.15)	9.05 a (±0.24)	9.23 a (±0.13)
10	9.01 a (±0.12)	9.10 a (±0.28)	9.40 a (±0.12)
11	8.71 a (±0.33)	9.36 a (±0.08)	9.44 a (±0.11)
12	9.08 b (±0.09)	9.75 a (±0.07)	9.59 a (±0.12)
13	8.76 b (±0.16)	9.63 a (±0.13)	9.96 a (±0.16)
14	9.27 a (±0.17)	9.86 a (±0.31)	10.2 a (±0.17)
15	9.25 b (±0.07)	9.57 ab (±0.10)	10.0 a (±0.16)
16	9.12 b (±0.07)	9.81 ab (±0.16)	10.2 a (±0.29)
17	9.45 b (±0.18)	9.96 ab (±0.08)	10.3 a (±0.20)
18	9.57 a (±0.20)	10.0 a (±0.16)	10.4 a (±0.21)
19	9.47 b (±0.15)	10.0 ab (±0.27)	10.5 a (±0.16)
20	9.57 a (±0.11)	10.0 ab (±0.19)	10.5 a (±0.16)

* Different lowercase letters within the column indicate a significant difference at *p* < 0.05 using Tukey’s test.

## Data Availability

Data are available from the authors upon request.
